# Uropathogenic *Escherichia coli* proliferate as a coccoid morphotype inside human host cells

**DOI:** 10.1371/journal.pbio.3003366

**Published:** 2025-09-03

**Authors:** Alaska Pokhrel, Lachlan Chisholm, Ariana Costas, Matthew Pittorino, Iain G. Duggin, Bill Söderström

**Affiliations:** 1 Australian Institute for Microbiology and Infection, University of Technology Sydney, Sydney, Ultimo, Australia; 2 Institut Cochin, INSERM U1016, CNRS UMR 8104, Paris, France; National Centre for Biological Sciences, INDIA

## Abstract

*Escherichia coli* is arguably one of the most studied bacterial model systems in modern biology. While *E. coli* are normally rod-shaped gram-negative bacteria, they are known to undergo conditional morphology changes under environmental and nutrient stress. In this study, using an infection-based in-vitro infection model system combined with advanced dynamical imaging, we present the first molecular details of uropathogenic *E. coli* (UPEC) dividing to form and proliferate as coccoid-shaped cells inside human host cells. For these intracellular UPEC cells, the frequency of cell division outpaced the rate of cell growth, resulting in a morphological transition from traditional rod-shape to coccobacilli. We also visualized the subcellular protein dynamics in these cells and noted that the division proteins follow the similar localization and constriction patterns that have been demonstrated for vegetative growth. However, unlike for fast-growing rod-shaped cells, FtsZ constriction in intracellular UPEC occurs prior to visual nucleoid segregation. Our results suggest that the modulation of division rate contributes to morphological adaptability of intracellular UPEC at the single-cell level.

## Abstract 2

Although typically rod-shaped, uropathogenic E. coli divide and proliferate as round coccoid cells during intracellular infection. This study shows that accelerated division via FtsZ constriction drives this transition, with the coccobacillus form persisting for generations.

## Introduction

Intracellular bacterial division and proliferation is essential for pathogenic bacteria to extend and maximize intracellular colonization during infection [1]. Division in the rod-shaped model bacterium *Escherichia coli* has been well mapped in laboratory settings, and is known to be carried out by a dynamic macromolecular complex, led by the highly conserved FtsZ protein [2]. FtsZ organizes an assembly of about 30 different proteins, collectively known as the ‘divisome’, which together coordinate the remodeling of the septum leading up to septation [3]. Guided by spatial regulatory systems (*e.g.*, the Min system [4] and Nucleoid occlusion [5], FtsZ is the first essential protein that localizes to midcell where it polymerizes into filaments forming an intermediate structure that resembles a ring, commonly referred to as the “Z-ring” [6]. Accumulation of FtsZ at the midcell also initiates the recruitment of other essential division proteins, of which FtsN is the last one to be recruited to the division site [7]. The arrival of FtsN to the thus far preassembled divisome complex is thought to trigger the initiation of septal cell wall synthesis; however, recent data from fast-growing cells indicate that the rate-limiting factor is the number of FtsZ molecules present in the Z-ring [8]. Recent advances in cryo-EM and machine learning have led to a better understanding of how the divisome is regulated on a molecular level. The initiation of septal cell wall synthesis through mechanisms of the FtsQLB complex, FtsI, and FtsN results in inward membrane invagination and eventually ring closure and cell separation [3,9–11]. *E. coli* cell division and morphology regulation during vegetative growth and antibiotic-induced stress conditions is well studied [12–14], and thoroughly reviewed elsewhere [15,16].

*E. coli* encompasses a versatile group of bacteria, which comprises of both harmless commensals as well as pathogens, with the ability to cause intestinal and extraintestinal diseases [17]. Due to their diversity in genomic and phenotypic context, it is one of the most used model organisms to study adaptation under various growth conditions, environmental niches, and infection settings. Depending on the site of infection, clinical pathogenic *E. coli* strains are divided into intestinal pathogenic *E. coli* and extraintestinal pathogenic *E. coli* (ExPEC) [17–19]. Uropathogenic *E. coli* (UPEC), the main causative agent for urinary tract infections (UTI), is one of the most common pathotypes that falls under ExPEC [17].

Nonpathogenic laboratory strains of *E. coli* have predominantly been used to investigate fundamental processes such as cell division and subcellular protein dynamics. As such, the proliferation of pathogenic *E. coli* (*e.g.*, UPEC) in host–pathogen intracellular infection settings remains significantly less well explored and understood. What is clear, however, is that during certain unfavorable environmental conditions, these bacteria modulate their growth and division as a survival strategy [20]. One striking example of this is the ability of rod-shaped bacteria to adopt both cocci and filamentous morphologies during UTI [21]. UTI are one of the strongest drivers of antimicrobial resistance globally [22], where UPEC accounts for approximately 75%–80% of all reported cases [23].

UPEC encode several virulence factors which enable them to successfully colonize and persist within the urinary tract, while evading the host defence mechanisms. These include cell surface-associated virulence factors (fimbriae, flagellum, capsular lipopolysaccharide, outer membrane proteins), secreted virulence factors (toxins, iron-acquisition mechanisms), and contains the traditional bacterial serotypes; the O-antigen, the K-Capsule, and the H-antigen [24–26]. Other factors involving metabolic flexibility also play an important role in the adaptability of UPEC to various microbiota, as UPEC typically originates in the gut before contaminating the periurethral area, and progressing into the bladder during UTI [27,28]. In this study, we have used *E. coli* strain UTI89, a prototype cystitis model strain to study UPEC proliferation in host cells. UTI89 was originally isolated from a patient with an acute bladder infection and belongs to the sequence type 95 (ST95) [27,29], and have serotype O18:K1:H7 [30].

The UPEC infection cycle during cystitis is largely understood in broad strokes [31], but few molecular details of the intracellular lifecycle of UPEC during UTI are currently known. An infection event is initiated when bacteria are internalized by superficial epithelial cells in the host bladder, where UPEC subsequently multiplies within the cytoplasm to form densely packed colonies known as intracellular bacterial communities (IBCs) [32,33]. Previous microscopy snapshots have indicated that some UPEC cells within dense IBCs in UTI murine models appear as cocci with cell lengths measuring around 1 μm or less [34,35]. Nutrient depletion was not considered a growth-limiting factor as the bacterial biomass increased over time [36], with generation times exceeding 60 min inside the bladder cells [34]. In a remarkable turn of morphology regulation, during the final step of the infection cycle, some UPEC cells grow into filamentous forms measuring hundreds of micrometres in length that subsequently flux out of the host cells into the bladder lumen [32]. These filaments would subsequently revert back to rods prior to invading surrounding cells and initiating a new round of infection [34].

While UPEC pathogenesis during UTI is quite well understood from a clinical perspective [24,25,37], single-cell division dynamics of UPEC inside the host cells has largely remained unexplored. Here, we followed the dynamics of bacterial proliferation and visualized subcellular protein localization (*e.g.*, fluorescently labeled FtsZ and FtsN) inside cultured human bladder cells, with the aim to understand molecular division dynamics that drive UPEC morphological adaptability at a single cell level. Our findings demonstrate that UPEC transitions from rod shape to a coccoid-like morphology very early in the infection, rather than only in dense, highly mature IBCs as previously reported [34]. Importantly, we also visualized the subcellular localization dynamics of division proteins and its regulatory systems (MinD, SlmA) in UPEC cells during host cell infection. In this environment, while the division proteins followed similar localization and constriction patterns as observed for rods in culture previously, FtsZ constriction appeared to be initiated prior to visual nucleoid segregation.

## Results

### Bacterial rod to coccobacilli shape transition during host cell infection

**Fig 1:**
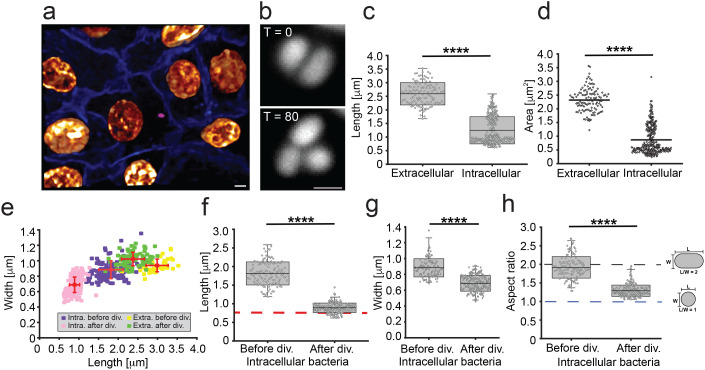
Intracellular UPEC actively divide into coccobacilli during UTI model infection. **a**, PD07i human epithelial bladder cells (membranes shown in blue [CellBrite Steady Membrane 405] and nuclei shown in gold [NucSpot Live Cell 650]) were challenged with fluorescently labeled UTI89 expressing cytoplasmic mCherry (pseudo coloured magenta). Imaged by confocal microscopy. Scale bar = 4 μm. **b**, Representative images of cells before (*T* = 0, start of imaging, 6-h post infection) and after division (*T* = 80); note that one daughter cell has left the field of view. Times (*T*) are shown in minutes. Images acquired using HiLo illumination. Scale bar = 1 μm. **c, d**, Average cell length (**c**) and area (**d**) of extracellular and intracellular bacteria (before any division event) (*n*_Intra_ = 326, *n*_Extra_ = 117). **e**, Length vs. width of extracellular (‘Extra’) and intracellular (‘Intra’) bacteria (before any division event). Error bars indicate S.D. **f–h**, Lengths (**f**), widths (**g**), and aspect ratio (**h**) of intracellular bacteria before and after division, (*n*_before_ = 126, *n*_after_ = 200). Red dotted line in **f** represents average coccobacilli width after division. Blue dotted line in **h** represents an aspect ratio of 1, i.e., a perfect circle. Box plots: box edge equals S.D., midline represents average, and whiskers indicate 1%–99% interval. Statistical significance was determined by student *T* test, where **** stars indicate p values less than 0.0001. The data underlying this figure can be found in S1 Data.

To follow intracellular UPEC proliferation over time, we used an in vitro infection model where a confluent layer of immortalized human epithelial PD07i bladder cells is challenged with the UPEC strain UTI89 (transformed to express a cytoplasmic marker, *i.e.*, mCherry) (Figs 1a and S1) [38,39]. The cells were subsequently incubated for 6 h to let bacteria invade the bladder cells and initiate intracellular proliferation. In order to visualize UTI89 proliferation inside PD07i cells post invasion, epithelial cell membrane and nucleus were stained with fluorescent dyes compatible with live cell imaging. Following 6-h postinfection, a time point commonly used in bladder infection studies [34], the division dynamics of bacterial cells were followed inside the bladder cells using live cell fluorescence microscopy (Fig 1). We observed UTI89 bacilli transition into a coccoid-like morphology with only one or a few cells present in the bladder cell cytoplasm (Fig 1b).

In agreement with the previous observations [34], we noticed that intracellular bacterial cells were overall significantly shorter than extracellular bacteria, measuring 1.25 ± 0.5 μm (*n*_Intra_ = 326) and 2.59 ± 0.41 μm (*n*_Extra_ = 117), respectively (Fig 1c), with an overall smaller area: Area_Intra_ 0.86 ± 0.51 μm^2^ (*n*_Intra_ = 326) versus Area_Extra_ 2.32 ± 0.43 μm^2^ (*n*_Extra_ = 117) (Figs 1d and S1 and S1 Movie). Following the division dynamics of intracellular bacteria over time (initial average length 1.81 ± 0.3 μm and width 0.88 ± 0.12 μm (*n* = 126) before division), we observed UTI89 rods actively dividing into coccobacilli shape with average length of 0.89 ± 0.13 μm and width of 0.68 ± 0.10 μm (*n* = 200) just after division (Fig 1e–1g), often with an aspect ratio close to 1 after division (average 1.29 ± 0.15, Fig 1h). The ratio of average length before and after division was ~ 2:1, indicating that the cells divided in half. In contrast, the average cell width was about 25% smaller after division indicating a shrinkage in the radial dimension. This observation is similar to previous reports of an overall reduction in size when bacteria are transferred from nutrient rich to nutrient poor media [40]. Overall, the intracellular measurements were significantly different from the apparent average cell length, width, and aspect ratio of extracellular cells (Figs 1 and S2). We also compared cell dimensions of intracellular cells with starved cells, grown to late stationary phase in either minimal (M9) or rich (LB) media. Starved cells were not as coccoid-shaped as the cells growing in the intracellular host environment, with the average aspect ratios of cells grown in M9 = 1.98 ± 0.52 μm and LB = 2.06 ± 0.55 μm, compared with intracellular coccobacilli cells = 1.29 ± 0.15 μm (S3 Fig).

When the division dynamics were followed over multiple generations, we observed that these intracellular coccobacilli divided into a new set of coccobacilli (S4 Fig and S2 Movie), suggesting that these bacteria maintain an altogether different size and morphological strategy compared to that in planktonic cultures where longer rod shapes are maintained.

Additionally, to determine whether this division behavior was more broadly adapted by UPEC, we challenged the bladder epithelial cells with another UTI-associated *E. coli* strain, MS2027 [41]. MS2027 was originally isolated from urine sample of a patient with nosocomial catheter associated UTI (CAUTI) [41]. Similar to UTI89, MS2027 encodes a range of virulence factors including fimbriae, capsular lipopolysaccharides, and toxins [41]. During intracellular bacterial proliferation, we observed that the division dynamics of MS2027 were indistinguishable from those of UTI89, with an average cell length before and after division measuring 1.64 ± 0.11 μm and 0.81 ± 0.08 μm, respectively (S5 Fig). Taken together, these data clearly show that intracellular UPEC cells actively divide into coccobacilli and maintain such morphology through multiple generations.

### Divisome dynamics of UPEC inside host bladder cells

**Fig 2:**
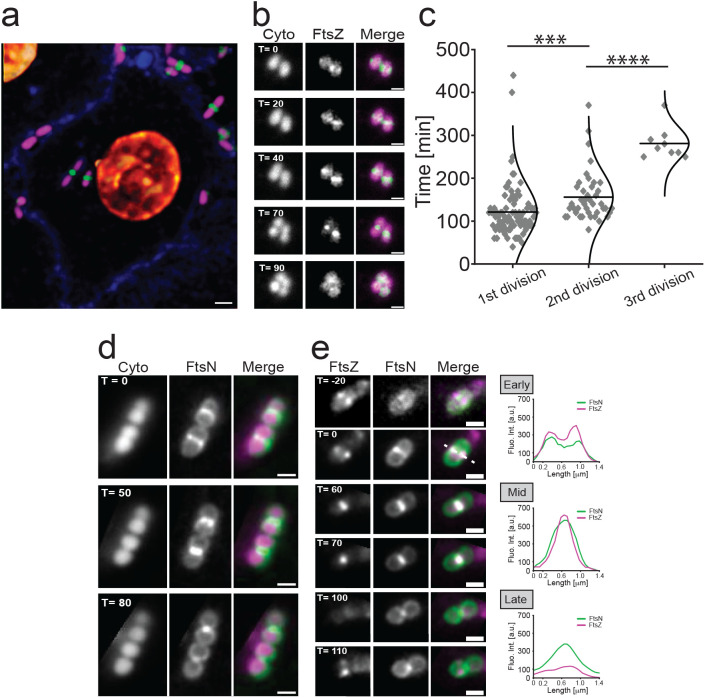
Divisome dynamics in intracellular UPEC cells during infection. **a**, Representative confocal image of intracellular UTI89 expressing a division marker (FtsZ-mCitrine; pseudo coloured green) with mCherry (pseudo coloured magenta) as cytoplasmic marker, bladder cell membranes shown in blue (CellBrite Steady Membrane 405) and nuclei shown in gold (NucSpot Live Cell 650). **b**, Still images of a time-lapse sequence showing FtsZ-mCitrine (pseudo coloured green) driven coccobacilli divisions imaged using HiLo illumination (S3 Movie). **c**, Average division times of intracellular bacteria over multiple generations. Midline represents average, *n*_first_ = 88, *n*_second _= 42, *n*_third _= 10. *** *p* = 0.0027. **** *p* < 0.0001. **d**, mCitrine-FtsN (pseudo coloured green) midcell constriction during coccobacilli divisions (S4 Movie). **e**, Subcellular FtsZ-mCherry (pseudo coloured magenta) and mCitrine-FtsN (pseudo coloured green) dynamics during coccobacilli divisions. Fluorescence intensity plots highlight a larger apparent radius of FtsN (Mid, *T* = 70) and that it remains longer at septum than FtsZ (Late, *T* = 100). Times (*T*) are shown in minutes since start of imaging (6 h p.i.). Scale bars **a** = 2 μm; **b**, **d** and **e** = 1 μm. The data underlying this figure can be found in S1 Data.

To explore the role of division proteins during the rod-coccobacilli transitions, we followed divisome dynamics in UTI89 expressing fluorescent division markers (FtsZ-mCitrine and/or mCitrine-FtsN) during intracellular divisions (Fig 2a, 2b, and 2d). FtsZ is the first essential division protein to arrive at the midcell, where it forms a ring-like structure called the Z-ring, which eventually constricts during division. By monitoring FtsZ accumulation at midcell over time, we followed Z-ring constriction until no detectable fluorescence signal remained at the septum. It has previously been shown that *ftsZ* is downregulated in intracellular UPEC during IBC growth compared to growth in liquid culture in rich media [42]. To minimize the influence of FtsZ over-expression in the experimental system, the level of FtsZ fluorescent fusion proteins in our set-up was less than 15% of total cellular amount (S6 Fig), which was previously shown to not interfere with overall FtsZ function and localization timing [8,43]. Here, intracellular coccobacilli interdivision times ranged from roughly 50 min up to more than two hours (Mean ± S.D.: 121.25 ± 61.6 min, *n* = 88), and beyond 6 h in an extreme case (Fig 2a–2c). Overall, the observed interdivision times were far greater than those of the UPEC cells grown in dishes in the tissue culture medium, but in the absence of bladder cells (S7 Fig). When intracellular coccobacilli proliferation was followed over multiple generations, we observed that the average division time since previous division generally increased, with the 2nd division taking 155.95 ± 57.72 min (*n* = 42) and 3rd division taking 281.11 ± 37.56 min (*n* = 10, note only few cells could be followed over multiple generations in this environment). The interdivision times also explored in MS2027, where FtsZ-mCitrine localization patterns and division dynamics mirrored those observed in UTI89, with an average division time of 165 ± 28 min (S5 Fig).

We also monitored FtsN, which is the last essential division protein to arrive at midcell [7] and signifies the start of septal peptidoglycan (cell wall) ingrowth that leads to membrane constriction and eventually daughter cell separation [3]. We followed the of midcell-accumulation mCitrine-FtsN in coccobacilli UPEC inside host cells over time, which indicated similar interdivision times to that of FtsZ in this environment (Fig 2d). Overall, the fluorescent fusions to both division proteins used here have previously been shown not to interfere with division or the growth of *E. coli* [39,43], and they are also highly likely to reflect the normal division process during intracellular infection (S6 Fig).

Additionally, we co-expressed FtsZ-mCherry and mCitrine-FtsN in UTI89 and followed the division dynamics over time in intracellular coccobacilli. Here, both these proteins followed the same overall patterns of localization as previously observed during division of rod-shaped cells, where FtsZ arrives at midcell prior to FtsN, and disassembles from the division site prior to FtsN toward the end of division [44] (Fig 2e and S5 Movie). Similarly, during active constriction, FtsZ-mCherry had apparent smaller radii compared to that of mCitrine-FtsN as seen in rods during vegetative growth and divisions (Fig 2e, “mid”) [45]. Combined, these data follow a clear trend suggesting that the divisome is involved in modulating the transition of intracellular rods to coccobacilli during infection, with key components of the divisome following similar localization dynamics as in vegetative growth [44].

### Division regulators implicated in bacterial filamentation and reversal do not appear to influence the transition to coccobacilli

We next investigated the roles of genes previously implicated in regulation of division during infection to see if they also play significant roles in bacterial morphology during the rod to coccobacilli transition. To this end, we followed the proliferation dynamics of UTI89 deletion mutants of *sulA* [46]*, *ymfM** [47], *and damX* [42] inside the PD07i bladder cells. The *sulA* and *ymfM* genes are induced during the DNA damage (SOS) response to pause division during DNA repair, although it is currently unclear whether these genes are significantly involved in bacterial morphology regulation during UTI [42,46,48,49]. In contrast, *damX* was found to be essential for blocking division during the infection-related filamentation (IRF) response in UTI, and additionally has been suggested to promote division during the normal cell cycle or when filaments revert back to rods after escape from host cells [39]; thus, it is possible that *damX* has other hypothetical roles in promoting UPEC division such as during coccobacilli formation. However, we found that UPEC strains with deletions of *sulA*, *ymfM*, or *damX* showed normal transitions from rod to coccobacilli during intracellular proliferation (S8 Fig). This suggests that the intracellular bacteria follow a different mode of cell shape regulation during intracellular proliferation that does not require these well-known regulators of bacterial division and filamentation.

### Min oscillations in intracellular coccobacilli

We next investigated the min system, a well-known positional regulator of rod-shaped cell division that is highly sensitive to cell size and shape. In rod-shaped *E. coli* cells, the division machinery is guided to midcell by the FtsZ-antagonistic effects of MinC, which is concentrated toward the cell poles [50,51]. This spatial regulation of MinC is achieved by the associated ATPase MinD and its activator MinE, which self-organize on membranes to create dynamic patterns that oscillate between the cell poles [52,53], resulting in a time-averaged concentration minima of MinCD at midcell [53–55].

**Fig 3:**
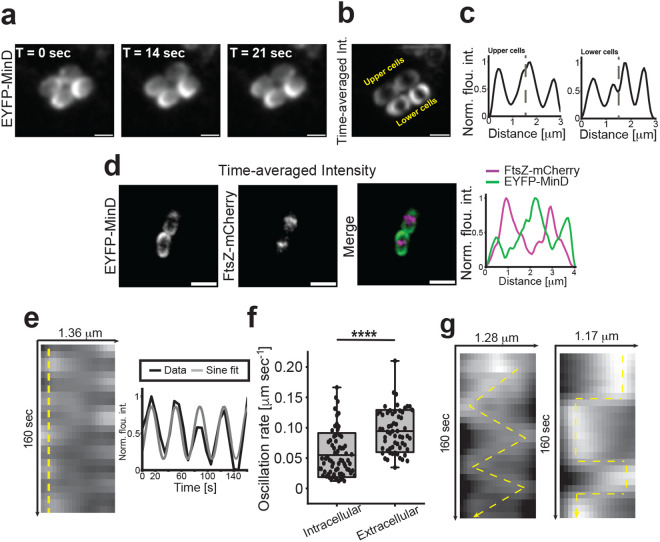
EYFP-MinD dynamics in UPEC coccobacilli during infection. **a**, Still images from an EYFP-MinD time-lapse series showing dynamics in intracellular UTI89 coccobacilli (S6 Movie). **b, c**, Time-averaged fluorescence intensity of EYFP-MinD. Dotted line in plots **(c)** represent cell boundaries. **d**, Time-averaged fluorescence intensity of EYFP-MinD (pseudo coloured green) and FtsZ-mCherry (pseudo coloured magenta), indicating that FtsZ localize at MinD minima also in coccobacilli. **e**, Representative kymograph of typical EYFP-MinD oscillations of a cell longer than 1.3 μm. Yellow dotted line represents where intensity plot was generated. **f**, Average EYFP-MinD oscillation rate in intracellular bacteria was 0.055 ± 0.036 μm s^−1^ (*n* = 71) and in extracellular 0.095 ± 0.035 μm s^−1^ (*n* = 60) cells. **g**, Typical kymographs of EYFP-MinD oscillations of cells shorter than 1.3 μm show complex nonstandard pole-to-pole oscillation dynamics. Kymographs in **g** were generated from the two lower cells in S6 Movie. Scale bars **a** and **b** = 1 μm; **d** = 2 μm. The data underlying this figure can be found in S1 Data.

Simulations and in-vitro experiments have suggested that short rods below a certain length (~2.5 μm or less) no longer undergo smooth MinD oscillations but instead show stochastic pole-to-pole switching or fail to generate patterns altogether [56,57]. We therefore examined EYFP-MinD dynamics in intracellular coccobacilli (Fig 3a), which revealed pole-to-pole oscillations and apparent circumferential movements along the cell perimeter, resulting in a time-averaged concentration minimum at midcell (Fig 3b and 3c, S6 Movie). Furthermore, in cells shorter than ~1.3 μm, we didn’t observe the typical pole-to-pole oscillations, but saw a much more complex EYFP-MinD dynamics, such as circular movements along the circumference and stochastic transitions from one pole to the other (Fig 3g). These cell sizes below which this irregular pattern was observed, is much smaller than the 2.5 μm previously predicted, further suggesting that coccobacilli have a distinct physiology from rod-shaped bacteria.

Like the localization patterns in rod-shaped cells, FtsZ-rings were observed at the EYFP-MinD concentration minima in coccobacilli, and always in the same plane as Z-rings from previous divisions, suggesting that a cell polarity is generally maintained in the coccobacilli (Fig 3d). MinD oscillation rates have previously been shown to depend on temperature, where a temperature shift from 20 to 40 °C resulted in 4-fold increase in oscillation patterns [58]. However, here at a constant temperature of 37 °C we found that the average EYFP-MinD oscillation speed normalized to cell length was significantly lower in intracellular bacteria: 0.055 μm s^−1^, compared to 0.094 μm s^−1^ for extracellular rod-shaped bacteria (Fig 3e and 3f). Our results indicate that the cell shape tightly linked to oscillation rate in abnormally short cells, and this could further be linked to previous observations from simulations suggesting that an increase in cellular concentration of the *min*-components lead to a reduction in oscillation rate [59].

### Chromosome segregation in intracellular UPEC coccobacilli

**Fig 4:**
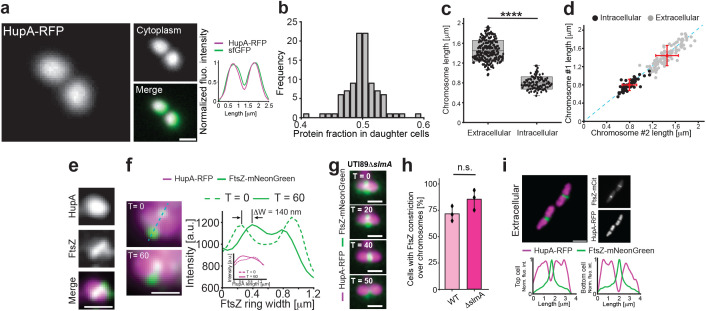
Chromosomal partitioning is maintained during intracellular UPEC coccobacilli divisions. **a, b**, DNA (HupA-RFP) partitioning in coccobacilli (msfGFP (green) as cytoplasmic marker). DNA segregates equally into daughter coccobacilli (*n* = 98). **c**, Average chromosome lengths in extracellular (*n* = 216) and intracellular bacteria (*n* = 98). **d**, Chromosomes partitions into equal size after division in both extracellular (gray) and intracellular (black) bacteria. Blue dashed line indicates linear dependence. Red dots indicate average, with *x*–*y* bars representing S.D. **e, f**, FtsZ-mNeonGreen (green) rings assemble and, partly, constrict over undivided chromosomes (HupA-RFP; psudo coloured magenta) in both WT and Δ*slmA* coccobacilli. Intensity plot in **f**, shows FtsZ constriction over 60 min (inset show HupA-RFP fluorescence profiles taken perpendicular to the Z-ring, with no intensity decrease at midcell over the same time interval). Cerulean dotted line indicate from where the FtsZ-mNeonGreen intensity plot is generated. **g**, Representative image sequence of UTI89Δ*slmA* expressing FtsZ-mNeonGreen (green) and HupA-RFP (pseudo coloured magenta) showing FtsZ-ring constriction over unsegregated chromosomes. **h**, Average levels of FtsZ-mNeonGreen constriction over unsegregated chromosomes from 3 independent infections (*n*_cell _= 30). **i**, FtsZ-mNeonGreen (green) rings do not constrict over unsegregated chromosomes (HupA-RFP, pseudo-coloured magenta) in extracellular rods. Statistical significance was determined by student *T* test, where **** stars indicate p values less than 0.0001., n.s. = not significant. Box plots: box edge equals S.D., midline represents average, and whiskers indicate 1%–99% interval. Times (*T*) are shown in minutes since start of imaging (6 h p.i.). Scale bars **a**, **e**, **f** and **g** = 1 μm; **i** = 2 μm. The data underlying this figure can be found in S1 Data.

Given that the intracellular coccobacilli measured only ~ 1/3rd of the length of typical pre-divisional rod-shaped cells, we then wondered how chromosomal partitioning was maintained during divisions of intracellular coccobacilli. To investigate this, we examined chromosome segregation dynamics of UPEC coccobacilli inside the host cells over time by expressing a DNA marker, HupA-RFP, which provides comparable nucleoid visualization as per the commonly used DAPI staining of nucleoids (S9 Fig and [60]). In coccobacilli, we never observed a cell division event where the DNA did not partition into both daughter cells (Fig 4a). Furthermore, DNA partitioning in coccobacilli was highly precise in dividing the DNA un equal parts into daughter cells (Fig 4b), and was indistinguishable to what has been found in rods previously [61]. Immediately after cell division, the average nucleoid length in intracellular coccobacilli measured 0.79 ± 0.12 μm (*n* = 100), while nucleoid length in extracellular rods measured 1.45 ± 0.2 μm (*n* = 216) (Fig 4c and 4d). Nucleoids in fast growing *E. coli* cells in rich media have been shown to be condensed, localizing close to the inner membrane. In cells treated with antibiotics (*e.g.,* mecillinam) that target the cell wall, forcing cells to adopt a round and wide shape, it was observed using single-molecule microscopy that this trend was enhanced. In contrast, nucleoids in slow growing cells, in nutrient poor media at lower temperature are localized at the radial center of the cells [62]. Possibly due to the reduced size of the intracellular coccoid shaped cells, their nucleoids exhibit localization patterns in between what has been observed previously, highly condensed and occupying most of the cytoplasmic space (Fig 4a). In fast growing cells it is common that the nucleoids split in two well before the previous division has concluded [63], however, this was never seen in the intracellular coccoid shaped cells with the limited resolution of the imaging approach in this study (Fig 4e).

The above results suggested that nucleoid segregation may substantially overlap with the midcell division machinery in coccobacilli. In order to determine if full chromosomal segregation occurs prior to initiation of Z-ring constriction, or if Z-rings would initiate constriction prior to visible completion of chromosome segregation in coccobacilli, we looked at DNA segregation (HupA-RFP) in relation to septal constriction (FtsZ-mNeonGreen) (S10 Fig). We observed that FtsZ-mNeonGreen readily formed bands over unsegregated nucleoids and appeared to initiate constriction prior to visible chromosome segregation in coccobacilli (Fig 4e and 4f); the FtsZ ring radius decreased up to ~140 nm (or roughly 1/3 of the diameter) prior to visible HupA-RFP intensity decrease at midcell (Fig 4f). Overall, this behavior was different to what was observed in extracellular bacteria during infection, where FtsZ-mNeonGreen constricted only when chromosomes were visibly separated (Fig 4i), even in short cells ~2–2.5 μm, just after previous divisions (S7 Movie).

DNA segregation in standard rod-shaped cells is regulated through interactions between the nucleoid and cell division machinery, including the nucleoid occlusion system [5]. In *E. coli*, the SlmA protein contributes to prevent FtsZ-ring constriction over unsegregated chromosomes, and this is achieved by complex interactions between DNA, SlmA, and the C-terminal binding domain of FtsZ [64,65]. Given the above results, we aimed to explore whether the nucleoid occlusion mechanism operates during intracellular rod-coccobacilli divisions. We therefore looked at Z-ring constriction in relation to DNA segregation in UTI89Δ*slmA* coccobacilli during intracellular divisions (Fig 4g). However, there was no statistically significant difference between the fractions of WT [85.3 ± 9.6%] and UTI89Δ*slmA* cells [72 ± 6.55%] initiating Z-ring constriction over nonsegregated nucleoids (Fig 4h). Previous results suggest that *slmA* is expressed by intracellular UPEC, so its role in chromosome segregation may be redundant or has as yet unknown consequences in regulation of standard rod and intracellular coccobacilli division and subsequently is down regulated during the recovery phases after bladder infection compared to intracellular expression levels, indicating a less central role of SlmA during intracellular growth and divisions [42,49].

## Discussion

Intracellular bacterial adaptation during host cell infection by UPEC suggested they undergo a morphological transition from rod to cocci during early infection. However, as this phenomenon was only captured in snapshot [34,36,66], the cellular and molecular dynamics underlying this transition remained largely unexplored. Here, using live cell fluorescence imaging, we show that the normally rod-shaped UPEC cells dynamically change their growth behavior to divide into coccobacilli during growth inside host cells. These morphological changes were initiated early, with only one or a few UPEC cells present within the bladder cells, and the UPEC cells maintained such morphology over multiple generations while in the host intracellular environment. This observation somewhat differs compared to a previous study that used a murine bladder infection models, where it was observed that coccobacilli formation was only observed after a period of intracellular growth as rods and in dense IBCs [34]. This suggests that UPEC are sensitive to initial intracellular conditions that differ between the fully differentiated mouse bladder epithelium [34] and the human immortalized cell line used here, which may provide future opportunities to identify factors that trigger the morphological transitions.

Coccobacilli divisions appear to be driven by regulation of UPEC cell division, with protein localization patterns and constriction dynamics remaining consistent with those observed during growth and divisions of rods in rich media, yet occurring overall earlier with respect to cell growth and elongation, resulting in the smaller coccobacilli daughter cells. In these cells, FtsZ assembled prior to division constriction, while FtsN arrived later at the division site at the onset of constriction. Furthermore, the overall molecular architecture appears consistent with rod cell division, with FtsN appearing outside the diameter of FtsZ during constriction. While our data shows FtsZ and FtsN localization patterns in intracellular coccobacilli during constriction are indistinguishable from those in rods, the overall interdivision times were noted to be on average six times slower than growth in rich medium.

Elongated growth along the long axis in bacilli, such as *E. coli*, is regulated and maintained by a number of proteins collectively known as ‘the elongasome’ [67]. Interestingly, during transitions into osmotic shock conditions in test tubes, the overall motion and rate of ‘elongation’ proteins remains the same as prior to the shock, resulting in a flat growth rate [68].

The cell division machinery and Min oscillation dynamics in coccoid *E. coli* cells have previously been investigated in mutant strains under pure culture conditions, where the coccoid cells lacking the Min proteins showed abnormal growth and cell division protein localization [57]. In this study, we co-produced fluorescently labeled FtsZ and MinD, and examined the intracellular protein dynamics in UPEC inside host cells. Here, the MinD dynamics were on average two times slower in coccobacilli than in extracellular rods, with pole-to-pole transitions switching from regular to irregular oscillations with decreasing cell size. The switching between these oscillation behaviors has previously been modeled to take place in cells measuring 2 μm or less in length, but here, we experimentally show this phenomenon takes place in coccobacilli cells that are considerably shorter than that, roughly ~1.3 μm in length. However, the average MinD concentrations were still maintained at a minimum at midcell in coccobacilli. The slower MinD pole-to-pole oscillations in coccobacilli could reflect the fact that the same amount of DNA has to be packed into a smaller cell volume without introducing partitioning errors, which could partly explain the overall prolonged coccobacilli division times. Previous simulation studies have noted that an increase in cellular concentration of the *min*-components has an influence on the oscillation rate [59], which correlates with our data and supports the idea that min-oscillation dynamics is dependent on cell dimensions.

A somewhat surprising observation in our experiments, and in contrast to what has been shown in fast-growing rods, was that FtsZ constriction appeared to be initiated prior to visual nucleoid segregation. While the underlying mechanisms behind this are not clear, we speculate that SlmA might have a less pronounced role during intracellular coccobacilli divisions since efficient chromosome partitioning was preserved in cells lacking *slmA* altogether. Z-rings have been observed to form over unsegregated nucleoids, guided to the midcell by the MatP protein in slow-growing *E. coli* cells, lacking both *slmA* and the *min* genes. However, it was not clear if the Z-rings initiated constriction at this stage of the division cycle [69]. Similarly, another study has reported Z-ring inhibition independent of the known nucleoid occlusion mechanism, when replication elongation was blocked by hydroxyurea or nalidixic acid [70]. However, in our study, we show initiation of FtsZ constriction prior to visual nucleoid segregation in intracellular coccobacilli.

On a cellular infection level, one plausible explanation for intracellular pathogens to divide into coccobacilli could be to allow for a higher number of bacteria per infected bladder cell, up to two orders of magnitude more for unorganized bacilli [71], or for a greater compaction of bacteria in IBCs, where UPEC cocci have shown a survival advantage over rods within intestine in murine models [35]. Thus, it is possible that division into coccobacilli with longer interdivision times during infection provides intracellular survival and persistence advantages [72].

The transcriptional profile of intracellular UPEC suggests that certain flagellar genes (e.g., *fliC* and *filL*) are upregulated in the first 4 h postinfection [73]. In addition to flagella genes, most genes that are upregulated are associated with various stress responses. The stationary-phase-associated gene, *dps* [74], implicated in the protection from various stresses including fatty acid starvation [75], was upregulated ~3-fold in the initial 4 h postinfection. Similarly, *pspA*, and other genes in the *psp* operon, were upregulated close to 1,000-fold compared to cells in vegetative growth. The PspA protein is known to interact with the shape maintenance protein, MreB [76], suggesting a possible role in cell shape modulating. Other stress response-associated genes that are activated during intracellular growth include *ibpA* and *ipbB*, both of which are part of a molecular chaperone family of proteins involved in promoting disaggregation of aggregated proteins [77]. Lastly, *bssS*, a gene implicated in increased biofilm formation in the mouse gut [78], was also upregulated, pointing toward an increased intracellular resilience of UPEC due to increased tolerance to oxidative stress [78].

Bacterial cells that experience rapid starvation generally show shrinkage of the cytoplasm and detachment of the inner membrane from the cell wall, especially at the new cells pole after division [79]. In the current study, the coccobacilli bacteria did not exhibit similar asymmetry.

It has been demonstrated that spatial confinement can induce significant changes in bacterial morphology [80,81], with remarkable robustness in division even in diverse shapes [60]. Bacteria are known to strive to maintain a stable surface-to-volume ratio, which plays a key role in maintaining a stable morphogenesis cycle [82]. However, the classical adder model of cell cycle regulation, where cells add a constant volume between birth and division [83–85], may not fully account for the UPEC divisions from rod-to-coccobacilli observed in this study due to the complex intracellular host environment. Moreover, the *E. coli* cytoplasm has been shown to shrink when moved from nutrient rich to nutrient poor media [79], with rapid response when exposed to osmotic shocks, but interestingly without altering the overall motion of cell shape determining components (*e.g.,* MreB) [68]. During intracellular growth of UPEC within IBCs, the actin-like MreB protein has been shown to be significantly upregulated [42]. MreB is required for rod shape in species such as *E. coli* through the action of MreB polymers, which orient perpendicular to the cell long axis and regulate the activity of cell wall synthases. Depletion of MreB results in cell rounding. Consequently, an increase in MreB levels in intracellular UPEC would be expected to promote rod shape instead of suppressing it. The fact that the opposite occurs suggests that perhaps too much MreB may phenocopy MreB depletion, similar to the properties of FtsZ.

A recent study revealed that the genetic requirements for UTI89 growth in M9-glycerol and intracellular growth in cultured bladder cells are largely shared [49]. Both of those conditions showed strong requirements of genes involved in macromolecular precursor biosynthesis and in the assimilation of nutrients, pointing to significant nutrient limitations for the bacterial cells while inside host intracellular environment [49]. Among the most important genes for intracellular survival, were *galU,* encoding a glucose-1-phosphate uridylyltransferase, responsible for the catalysation of UDP-d-glucose from UTP and α-d-glucose 1-phosphate, and *pgm* (phosphoglucomutase), encoding an enzyme involved in converting α-d-glucose 6-phosphate to α-d-glucose 1-phosphate. Similarly, the glucosyltransferase encoded by *opgH*, a UDP-glucose-activated cell division regulator which directly binds to FtsZ inhibiting and/or delaying cell division [86], was also implicated in survival but to a much lesser degree [49]. Deletions of either *galU*, *pgm,* or *opgH* were all shown to statistically reduce the size of nonpathogenic *E. coli* grown in LB [86]. Thus, it is possible that differential regulation of *galU* in the context of intracellular UPEC coccoid divisions could lead to regulatory changes in the *opgH*-pathway, and thus in overall division rates in intracellular UPEC during host invasion.

A separate study reported a similar phenomenon, where growth of UPEC under spatial confinement in a microfluidics system resulted in a shortening phenotype [81]. This observed morphology was linked to modifications in the physical properties of the cells, driven by transcriptional shifts upon confinement. Specifically, the Rcs pathway was suggested to be of significance, as it facilitates cell envelope re-modeling to withstand high turgor pressure. Notably, *rcsA and rcsC* may be important during intracellular proliferation of UPEC [49]. Thus, preserving bacterial shape during confinement both in vitro and in host cells could share common regulatory functions [81].

In summary, the intracellular UPEC cells in our study not only actively divided into coccobacilli, they also showed a slight reduction in width, pointing to a possible nutrition limitation that affects the length and width of UPEC while inside the host cells [40]. Taken together, our data support the idea that the morphological transition of UPEC from rods to coccobacilli occurs through regulation of machineries involved in division and cell width, and may serve as a survival strategy. This UPEC response to the host cell might be triggered by a combination of low nutrient availability [28,40] and mechanical confinement [81], both of which appear to be present in the hostile, crowded environment encountered during intracellular life in host cells.

## Methods

### Plasmid engineering

To engineer pMP7, the *ftsZ-mNG* sandwich fusion, originally sourced from BW27783/FtsZ-55mNeonGreen56 [87] (gift from Harold Erickson, Duke University), was PCR amplified from plasmid pDD3 (gift from Daniel Daley, Stockholm University) using primers 1 and 2. The vector backbone, pGI3, was digested with *NcoI* and *BamHI*. Both DNA fragments were then purified and ligated by *in vivo* DNA synthesis. To make pMP11, *mCherry* was PCR amplified from pDSW931 [88] (Gift from David Weiss, University of Iowa) using primers 3 and 4, and the vector, pMP7, was amplified using primers 5 and 6 (all primers are listed in the supplementary document. Both DNA fragments were then purified and ligated by *in vivo* DNA synthesis. The sequences were verified by Sanger sequencing (AGRF, Australia).

### Bacteria growth and protein production

Bacterial strains used throughout the study were routinely plated on LB agar (Difco) plates supplemented with appropriate antibiotics (Ampicillin: 100 μg ml^−1^ (Sigma), Spectinomycin 100 μg ml^−1^ (Sigma), Kanamycin 50 μg ml^−1^ (Sigma)). A single colony of respective *E. coli* strain was grown in LB media (Difco) or M9 minimal media (1× M9 salts, 0.1% glucose, 0.1% casamino acid, 0.01 M MgSO_4_, 1 μg ml^−1^ nicotinamide) supplemented with appropriate antibiotics at 37 °C overnight without shaking. Following overnight growth, cultures were were pelleted and resuspended in 1× PBS (Bio-Rad) to an optical density (OD_600_) of 0.1. Fluorescent protein fusion production was initiated by addition of 1 μM IPTG for FtsZ-mCitrine (pHC054 [45]) in UTI89 (2 μM IPTG in MS2027), 5 μM IPTG for EYFP-MinD (pSR-4 [61]) and HupA-RFP (pSTC011 [89]), and 15 μM Rhamnose for mCitrine-FtsN (pHC004 [90]). Note that FtsZ-mNeonGreen (pMP7), FtsZ-mCherry (pMP11), msfGFP (pGI5 [38]) and mCherry (pGI6 [91]) did not require induction as they are produced under constitutive promoters. Cell viability, generation times, and cell dimensions were comparable to those of a WT strain grown under the same conditions (S6 Fig). Bacterial strains and plasmids used in this study are listed in S1 Table.

*Primers used in this study:*

1. GATAGCGCCCGGTCTAGAGGAGGTACTACCATGTTTGAACCAATGGAACTTACC
2. GGCTGCAGGTCGACGGATCTTTAGGATCCTTAATCAGCTTGCTTACGCAGG
3. GGTTGGAGGATCCACCCTCGAGATGGTGAGCAAGGGCGAGGAG
4. GAATCGTCTGGGTGGATCCCTCGAGCTTGTACAGCTCGTCCATGCCG
5. CTCGAGGGATCCACCCAGACGATTCAAATC
6. CATCTCGAGGGTGGATCCTCCAACCG

### Cell culture

Immortalized human epithelial bladder cells PD07i [92] were maintained in antibiotic-free growth EpiLife Medium (Gibco) supplemented with growth supplement (HKGS) at 37 °C in the presence of 5% CO_2_. Cells were passaged using standard trypsinization protocols upon reaching ~ 75%–90% confluency. To prepare for infection, cells were seeded in 35 mm glass-bottom petri-dishes (IBIDI, #1.5H glass, Cat. No:81158) and grown to confluency.

### Infection model

Seeded petri-dishes were challenged with the appropriate bacterial strain at an MOI of ~100 (roughly corresponding to 10^7^ cells as determined by CFU counts). To facilitate increased bacterial adherence to the bladder cells, dishes were centrifuged at 500 rpm for 3 min, before incubation at 37 °C (5% CO_2_) with orbital agitation (50 rpm) for 15 min. Media containing bacteria was removed and the petri-dishes were replenished with fresh pre-warmed EpiLife media supplemented with inducer as appropriate. Dishes were incubated for at least 6 h to let bacteria invade the bladder cells and initiate intracellular divisions (which coincides with “middle IBC” maturation [34]). Following incubation, media was removed from petri-dish and cells were washed once with 1 × PBS. The bladder cells were stained for membrane (CellBrite Steady Membrane 405) and nucleus (NucSpot Live Cell 650) for 30 min in pre-warmed EpiLife as per manufacturer’s instructions (Biotium, San Francisco, USA). After the staining step and two washes with 1 × PBS, cells were covered in fresh pre-warmed EpiLife (supplemented with inducer as required) and Gentamicin f.c. Fifty μg ml^−1^ (Sigma) and taken for imaging. Only intracellular bacteria survive this standard Gentamicin protection assay treatment [93], and thus dividing bacteria are inside bladder cells (S1 Movie) while nondividing bacteria are extracellular (S8 Movie). For DAPI staining, extracellular UTI89 cells expressing HupA-RFP were incubated with Hoechst (Invitrogen, H33342, 1:1000) for 10 min before being imaged. The infected PD07i cells were permeabilised with 1% Triton X-100 (Sigma, X100-500 ml) before ProLong Diamond Antifade Mountant with DAPI (Invitrogen, P36962) was added and imaged. Generally, all infection experiments were run in at least three independent replicates, unless otherwise indicated.

### Imaging

For time-lapse imaging, live cells were imaged in the glass-bottom petri dishes on a Nikon N-STORM v5 TiE2 (with NIS v.5.30) microscope in TIRF mode using HILO illumination, with a 100 × 1.49 NA oil objective, to increase the signal-to-noise ratio. To support live cell imaging, the microscope was equipped with a stage top environmental chamber regulating temperature (37 °C) and CO_2_ levels (5% CO_2_). 405, 488, 561, and 647 nm laser lines were used to excite fluorescent probes (405: membrane dye. 488: mCitrine, mNeonGreen, EYFP. 561: mCherry. 647: nuclear dye) as appropriate. Prior to imaging the bladder cells, cells were scanned in the Z-direction to make sure that the bacteria expressing pGI6 in the relevant XY-point were lying down (relevant to Fig 1). For bacteria also expressing fluorescent protein fusions to cell division proteins this was not needed as a ring (e.g., FtsZ-mCit) was clearly visible in bacteria lying down in the imaging plane. Acquisition times were 70–400 ms and image size 2048 × 2048 (pixel size 65 nm). Fluorescence emission was collected via emission filters sets for DAPI, FITC, TxRed and ‘Normal STORM (647)’. Images were captured using a sCMOS Flash 4.0 v3 (Hamamatsu) camera. To follow the bacterial cell division within live bladder cells, images were acquired every 7–10 min for at least 6 hours (Note: it was generally not possible to accurately track more than 1 division event due to the intracellular movements with bacteria transitioning in and out of focus). To follow short term EYFP-MinD dynamics, images were acquired every 5–7 s for at least 2 min.

For confocal live cell imaging, images were acquired on a Leica Stellaris 8 confocal microscope equipped with a 63× NA1.49 oil objective enclosed in an environmental chamber at 37 °C in the presence of 5% CO_2_. Fluorophores were excited using a white laser, and emission was collected using software-optimized settings in the LAS software. Image size was chosen to either 2,048 × 2,048 or 4,096 × 4,096 pixels, with individual pixel size 90 and 45 nm, respectively. The pinhole was set to 1 AU and Z-stacks were collected with step length of either 125 or 250 nm (15–99 images per stack, depending on step length and thickness of the bladder cell in question).

### Image postprocessing

Raw microscopy images were transferred to Fiji for processing and analysis. Time-lapse image sequences (both for overnight division dynamics and for MinD-FP dynamics) were drift-corrected using StackReg, or HyperStackreg where appropriate, and background was subtracted using a rolling ball radius of 50. Images were denoised using the PureDenoise plug-in. To follow division of intracellular bacteria (Fig 1b–1g), time-lapse sequences were segmented based on cytoplasmic fluorescent protein intensity using the tracking workflow in the ilastik Fiji plug-in (version 1.4.0). Divisions were determined being over at the time point of the first image of visually separated cells. EYFP-MinD-based kymographs were generated using KymoResliceWide. Time-averaged fluorescence intensity images of EYFP-MinD were generated using the standard deviation option in the Z-projection tab. Deconvolution and reconstruction of confocal Z-stacks were performed using Leica LAS.

### Data analysis and statistics

Cell length, area, aspect ratio, and chromosome length of intra- and extracellular cells (rods and coccobacilli) were extracted by using the MicrobeJ [94] plug-in or occasionally from manually traced dimensions in Fiji. Extracellular bacterial cells that were clearly located outside of and away from the PD07i cells (based on cytoplasmic membrane staining) were only used for data collection for “extracellular cells”. Intracellular UPEC expressing fluorescent protein fusions to a division protein were followed for 1–3 divisions in time-lapse movies to manually extract intracellular division times. Visible FtsZ-FP accumulation at midcell was considered as initiation of division, and its disassembly from midcell was considered as completed division. Generally, fluorescence intensity profiles were generated in Fiji and analyzed in Origin Pro 2021 Academic (V9.8.0.200). EYFP-MinD oscillation rates per length in intra- and extra-cellular cells were determined by measuring cell lengths and extracting fluorescence profiles over time generating corresponding kymographs. Statistical analyses were performed in either Origin Pro 2021 Academic or GraphPad Prism 10 (V10.0.3). Statistical significance was determined by student *T* test, where **** stars indicate *p* values less than 0.0001, unless defined otherwise. Figures were prepared using Adobe Illustrator.

### Western blotting

A culture volume corresponding to 0.1 OD units was collected from each UPEC strain expressing the protein of interest after at least 6 h of growth in PD07i-seeded petri-dishes (S6 Fig). The samples were pelleted and re-suspended in SDS-loading buffer and resolved by SDS-PAGE gel electrophoresis. Proteins of interest (FtsZ, FtsN, and MinD) were transferred to nitrocellulose membranes using a semi-dry Transfer-Blot apparatus (Bio-Rad). The Nitrocellulose membranes were then blocked in 5% (w/v) skim milk and probed with the respective primary (anti-FtsZ, Agrisera. Anti-FtsN [95]. Anti-MinD antisera, gift from Dr. Yu-ling Shih, Academia Sinica, Taiwan). All primary antibodies were used at 1:3333 and secondary antibody (anti-rabbit HRP, 1:10000, Bio- Rad) before being washed and imaged (GE Amersham Imager 600).

## Supporting information

S1 Fig Infected PD07i bladder cells imaged by confocal microscopy. Nucleus (gold) and membrane (blue). Right side shows images without the membrane channel for better visibility of intracellular bacteria. **a**, A bladder cell that has taken up UTI89 (Cytoplasmic mCherry: pseudo coloured magenta). **b**, A bladder cell in the process of taking up UPEC, red arrows (Cytoplasmic mCherry: pseudo coloured magenta, FtsZ-mCitrine: pseudo coloured green). **c, d**, A cell where some UTI89 have been taken up (bacterial cells dimmed by the membrane), while others that are external (cells not dimmed by the membrane). Scale bars 4 µm.
(PNG)

S2 Fig Aspect ratio (length/width) of intracellular UTI89 (mCherry) within PD07i bladder cells, and extracellular UTI89 in EpiLife growth media, before and after divisions. Four **** (*p* > 0.0001) indicate extremely statistically significant difference. “Intra” = intracellular bacteria, “Extra” = extracellular bacteria. Box plots: midline indicates mean, and box represents S.D. Whiskers encompass 199% interval of the data. The data underlying this figure can be found in S1 Data.
(PNG)

S3 Fig UTI89 cell dimensions when starved (grown to late stationary phase) compared with the intracellular bacteria after division. Representative images of UTI89 cells grown to late stationary phase (24 h) in M9 minimal media (**a**) and LB media (**b**). Scale bar 5 µm. Cell length (**c**), width (**d**) and aspect ratio (**e**) of UTI89 cells grown in M9 or LB for 24 h (*n* = 224) compared with intracellular UTI89 within PD07i bladder cells after division (*n* = 200). Four **** (*p* > 0.0001) indicate extremely statistically significant difference. The data underlying this figure can be found in S1 Data.
(PNG)

S4 Fig Still images from a time-lapse image sequence (S2 Movie) showing UTI89 coccobacilli-to-coccobacilli divisions inside host bladder cells. The UTI89 strain expresses cytoplasmic mCherry (pGI6), pseudo coloured gray. Scale bar 1 µm.
(PNG)

S5 Fig Clinical UTI isolate MS2027 follows the same rod-to-coccobacilli division patterns inside host cells as UTI89. **a**, Representative images from a time-lapse sequence of PD07i human epithelial bladder cells (membranes in blue) challenged with MS2027 expressing mCherry (pseudo colored magenta) in the cytoplasm; cells before (*T* = 0) and after division (*T* = 200). **b**, Images of intracellular MS2027 cells before and after division across different PD07i human epithelial bladder cells from three separate infections. Example cell lengths of intracellular MS2027 cells before (rods) and after division (coccobacilli) are noted in the table. **c**, Still images from a time-lapse sequence of intracellular MS2027 cells expressing FtsZ-mCitrine (pseudo coloured green), and mCherry in the cytoplasm. Times (*T*) are shown in minutes. Scale bars: **a** = 5 µm (insets 1 µm), **b** = 0.5 µm and **c** = 2 µm.
(PNG)

S6 Fig Bacterial strain viability and quantification of fluorescent protein fusion expression levels. **a,** Growth curves of UTI89 strains used in the study over a 6h growth period in EpiLife media. **b, c**, Average cell lengths (**b**) and widths (**c**) of UTI89 strains grown in EpiLife media in petri-dishes at 37 °C in the presence of 5% CO_2_. **d**, Growth curves of MS2027 strains used in the study over a 6 h period in EpiLife media. **e, f**, Average cell length (**e**) and width (**f**) of MS2027 strains grown in EpiLifemedia in petri-dishes EpiLife media at 37 °C in the presence of 5% CO_2_. All growth curves include data from 3 biological replicates and *n*_cell_ = 300 for lengths and widths measurements. Midline represents average, and whiskers indicate S.D. **g–I,** Quantitative western blots of FtsZ-mCitrine, FtsZ-mCherry, FtsZ-mNeonGreen, mCitrine-FtsN, and EYFP-MinD production levels in UTI89 and MS2027 cells. FtsZ-mCitrine in (**g**) UTI89, and in (**I**) MS2027 was produced at 13.5(±5.8) % and 7.2(±3.1) % of the total cellular FtsZ, respectively. (**h**) FtsZ-mCherry in the UTI89 + mCitrine-FtsN and in UTI89 + EYFP-MinD strains was produced at 16.8(±8.3) % and 18.5(±7.9) % of the total cellular FtsZ respectively. **(i)** FtsZ-mNeonGreen in the UTI89 + HupA-RFP and in UTI89Δ**slmA* *+ HupA-RFP strains was produced to 18(±2.6) % and 17.4(±3.1) % of the total FtsZ. (**j**) mCitrine-FtsN in UTI89 + mCh_CYTO_ and UTI89 + FtsZ-mCherry strains was produced to approximately 142(±14.5) % and 118.7(±1.5) % of the native FtsN production, respectively. Overall, these values for FP-FtsN and FtsZ-FP have previously been shown not interfere with growth and division during vegetative growth in rich media. (**k**) EYFP-MinD production in the UTI89 + FtsZ-mCherry strain showed that EYFPMinD was produced at approximately 28(±6.4) % of the total cellular MinD. Note some unspecific binding of the anti-MinD antisera. Collectively, these results show that the fluorescent protein fusions were not heavily overexpressed in any of the strains used in this study. All percentages (± S.d.) are based on three independent experiments. mCh, mCherry; mCit, mCitrine; mNG, mNeonGreen; GFP_CYTO_, Free GFP in the cytoplasm; mCh_CYTO_, Free mCherry in the cytoplasm. The data underlying this figure can be found in S1 Data.
(PNG)

S7 Fig Growth of the WT UTI89 strain in EpiLifemedia at 37 °C in the presence of 5% CO_2_. Average doubling time: 31 ± 1 min (*n* = 3). The data underlying this figure can be found in S1 Data.
(PNG)

S8 Fig Deletion of genes previously implicated in morphology regulation during infection does not affect rod-to-coccobacilli divisions. Representative images of PD07i human epithelial bladder cells (membrane in blue) challenged with **a**, WT UTI89, **b**, UTI89Δ*damX,* **c**, UTI89Δ*sulA*, and **d,** UTI89Δ*sulA*Δ*ymfM.* All strains expressed mCherry from pGI6 in the cytoplasm as a volume marker (pseudo-coloured magenta). Example coccobacilli lengths of parental UTI89 and mutant strains after division are noted in the insets. Scale bars **a–d** = 5 µm (insets 1 µm).
(PNG)

S9 Fig HupA-RFP signal overlaps with DAPI signal in extracellular (a, b) and intracellular UTI89 cells (c, d). Representative images of nondividing (upper panel) and dividing (lower panel) extracellular UTI89 cells stained with DAPI (blue) and expressing HupA-RFP (pseudo-coloured magenta) (**b**), and PD07i human epithelial bladder cells challenged with UTI89 expressing HupA-RFP (**d**). DAPI stained nucleus and intracellular UTI89 cells are shown in gray (pseudo-coloured), and HupA-RFP expression in magenta (pseudo-coloured). *n* = 103 (**a**), *n* = 14 (**c**). Scale bars (**b**) = 1 µm, (**d**) = 2 µm. The data underlying this figure can be found in S1 Data.
(PNG)

S10 Fig Representative microscopy image using HiLo illumination of PD07i bladder cells challenged with UTI89/HupA-RFP/FtsZ-mNeonGreen. PD07i membranes in blue, nuclei in gray, HupA (pseudo coloured magenta) and FtsZ in green. Scale bar 20 µm.
(PNG)

S1 Table Bacterial strains and plasmids used in the study. (PDF)

S1 Movie Time-lapse of a bladder cell (membrane in gray) invaded by UTI89 (mCherry pseudocoloured magenta). Gentamicin protection assay, f.c. 50 µg ml^−1^. Note that only intracellular bacteria are viable. Imaged using HILO illumination. Scale bar 2 µm.
(AVI)

S2 Movie Time-lapse of UTI89 coccobacilli to coccobacilli divisions (mCherry pseudo-coloured gray). Imaged using HILO illumination. Scale bar 1 µm.
(AVI)

S3 Movie Time-lapse of intracellular UTI89 (mCherry pseudo-coloured magenta) expressing FtsZ-mCitrine (green). Note at *T* = 90 min that FtsZ-mCitrine rings have disassembled. Imaged using HILO illumination. Scale bar 1 µm.
(AVI)

S4 Movie Time-lapse of intracellular UTI89 (mCherry pseudo-coloured magenta) expressing mCitrine-FtsN (green). Imaged using HILO illumination. Scale bar 1 µm.
(AVI)

S5 Movie Time-lapse of intracellular UTI89 expressing FtsZ-mCherry (left) and mCitrine-FtsN (mid). Merged channels (right) FtsZ-mCherry (magenta) and mCitrine-FtsN (green). Imaged using HILO illumination. Scale bar 1 µm.
(AVI)

S6 Movie Time-lapse of intracellular UTI89 expressing EYFP-MinD. Imaged using HILO illumination. Scale bar 1 µm.
(AVI)

S7 Movie Time-lapse of extracellular UTI89 expressing HupA-RFP (left) and FtsZ-mNeonGreen (mid) and merged (HupA-RFP in magenta, mNeonGreen in green). Prior to addition of Gentamicin. Imaged using HILO illumination. Scale bar 4 µm.
(AVI)

S8 Movie Time-lapse of extracellular UTI89 expressing HupA-RFP (magenta) and FtsZ-mNeonGreen (green) with 50 µg ml^−1^ Gentamicin added to the EpiLife growth media. The chromosomes are condensed to midcell and FtsZ rings are disassembled. Time between Gentamicin addition to media in the dish and imaging <10 min. Imaged using HILO illumination. Scale bar 4 µm.
(AVI)

S1 Data Raw data used for plots in this study. (XLSX)

S1 Raw Images Uncropped western blots. (PDF)

## Abbreviations

ExPEC: extraintestinal pathogenic *E. coli*
IBCs: intracellular bacterial communities
IRF: infection-related filamentation
UPEC: uropathogenic *E. coli*
UTI: urinary tract infections
