# Supplementary figures and images for: Uropathogenic Escherichia coli proliferate as a coccoid morphotype inside human host cells
Source: PLoS Biol. 2025 Sep 3;23(9):e3003366. doi: 10.1371/journal.pbio.3003366 (PMC12407437; doi:10.1371/journal.pbio.3003366)

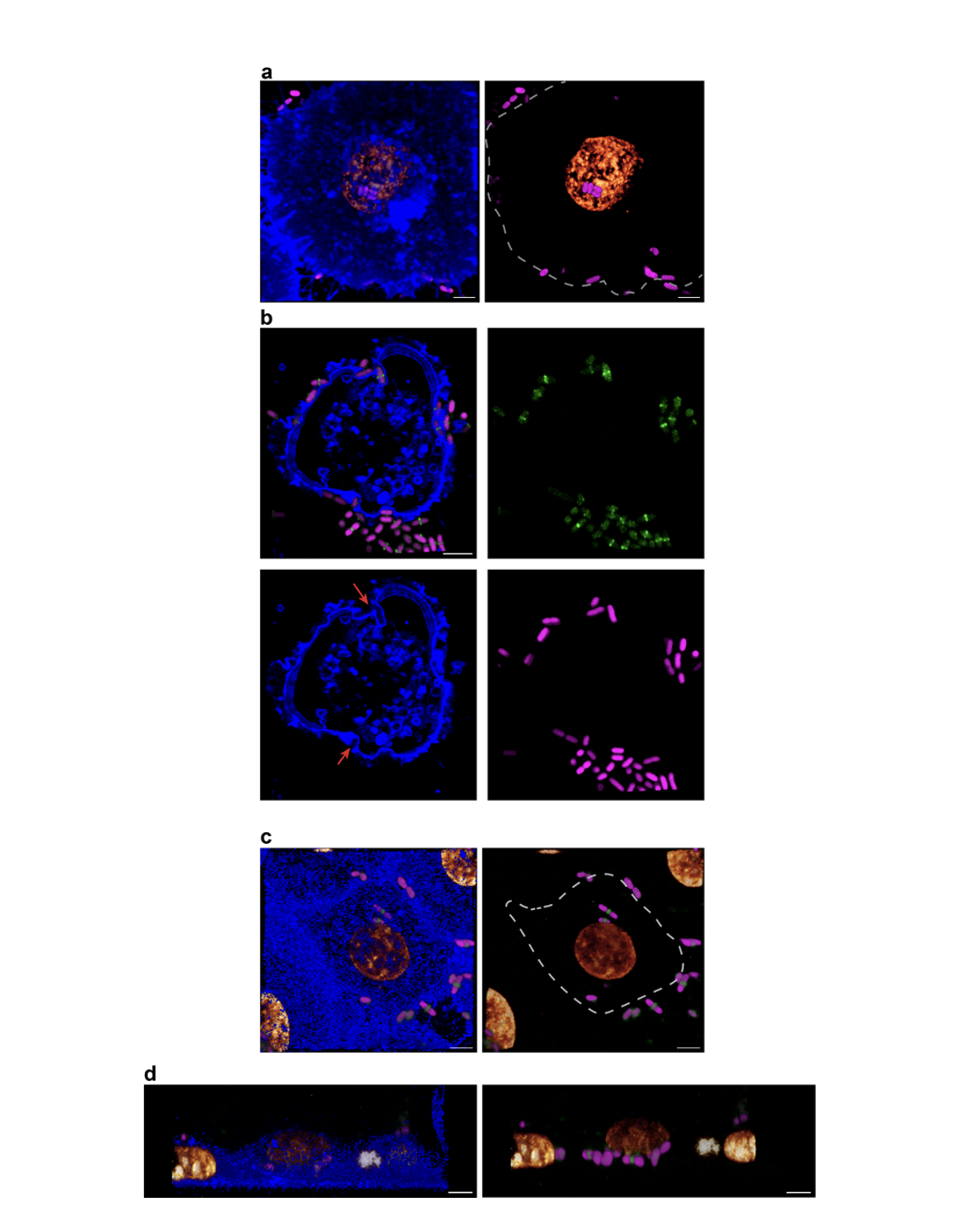

Supplement: S1 Fig — Nucleus (gold) and membrane (blue). Right side shows images without the membrane channel for better visibility of intracellular bacteria. a, A bladder cell that has taken up UTI89 (Cytoplasmic mCherry: pseudo coloured magenta). b, A bladder cell in the process of taking up UPEC, red arrows (Cytoplasmic mCherry: pseudo coloured magenta, FtsZ-mCitrine: pseudo coloured green). c, d, A cell where some UTI89 have been taken up (bacterial cells dimmed by the membrane), while others that are external (cells not dimmed by the membrane). Scale bars 4 µm. (PNG) [file pbio.3003366.s001.png]

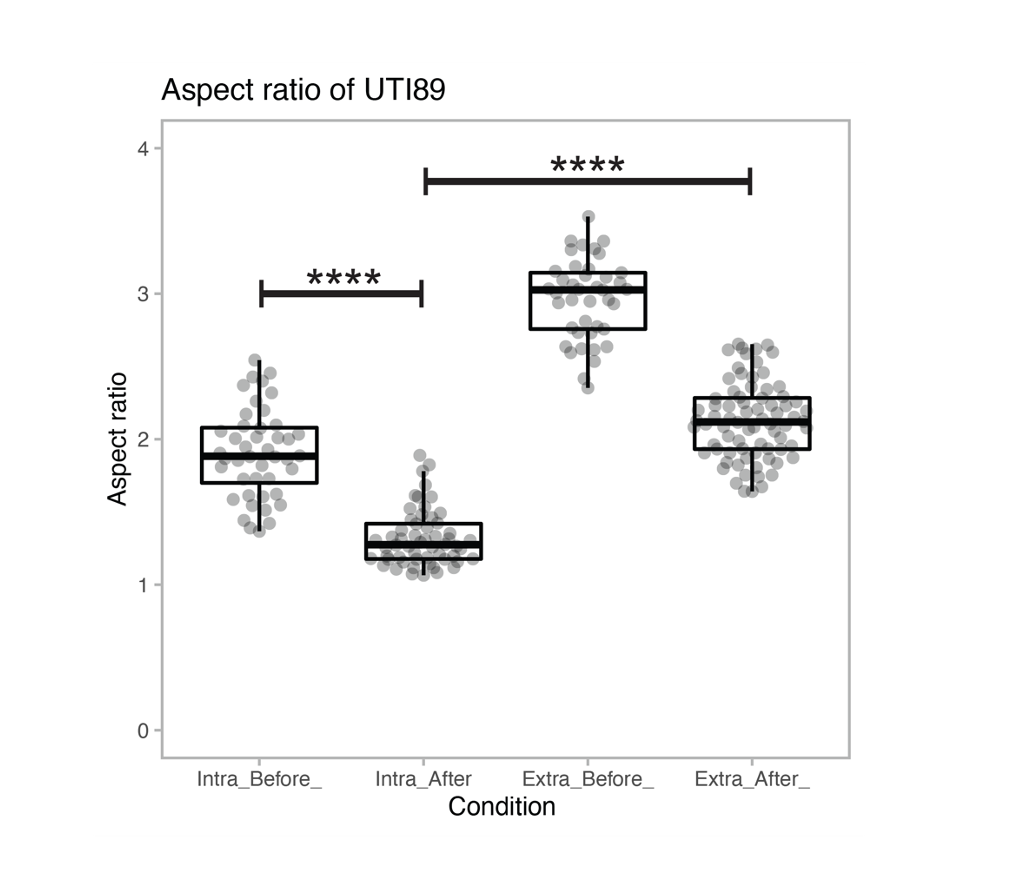

Supplement: S2 Fig — Four **** (p > 0.0001) indicate extremely statistically significant difference. “Intra” = intracellular bacteria, “Extra” = extracellular bacteria. Box plots: midline indicates mean, and box represents S.D. Whiskers encompass 199% interval of the data. The data underlying this figure can be found in S1 Data. (PNG) [file pbio.3003366.s002.png]

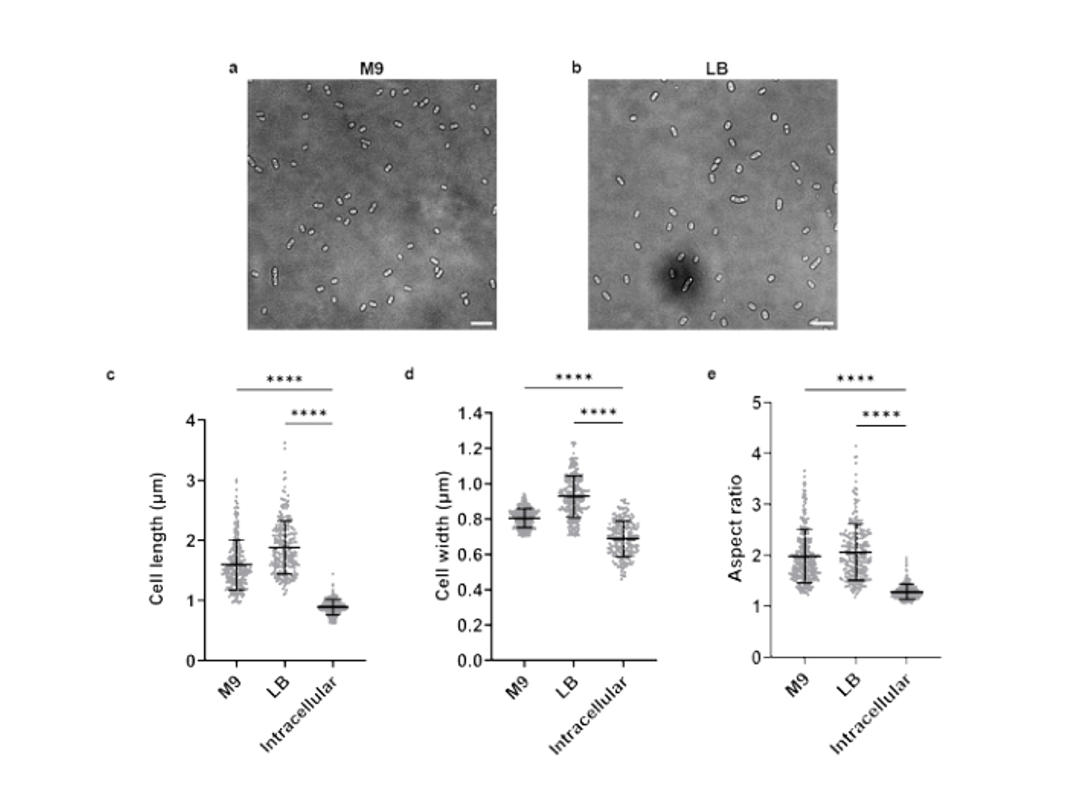

Supplement: S3 Fig — Representative images of UTI89 cells grown to late stationary phase (24 h) in M9 minimal media (a) and LB media (b). Scale bar 5 µm. Cell length (c), width (d) and aspect ratio (e) of UTI89 cells grown in M9 or LB for 24 h (n = 224) compared with intracellular UTI89 within PD07i bladder cells after division (n = 200). Four **** (p > 0.0001) indicate extremely statistically significant difference. The data underlying this figure can be found in S1 Data. (PNG) [file pbio.3003366.s003.png]

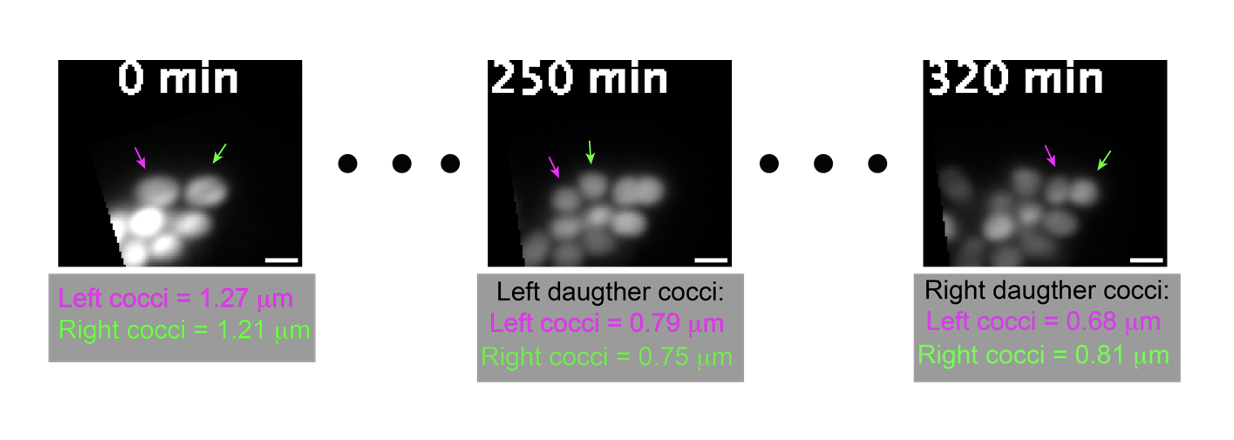

Supplement: S4 Fig — The UTI89 strain expresses cytoplasmic mCherry (pGI6), pseudo coloured gray. Scale bar 1 µm. (PNG) [file pbio.3003366.s004.png]

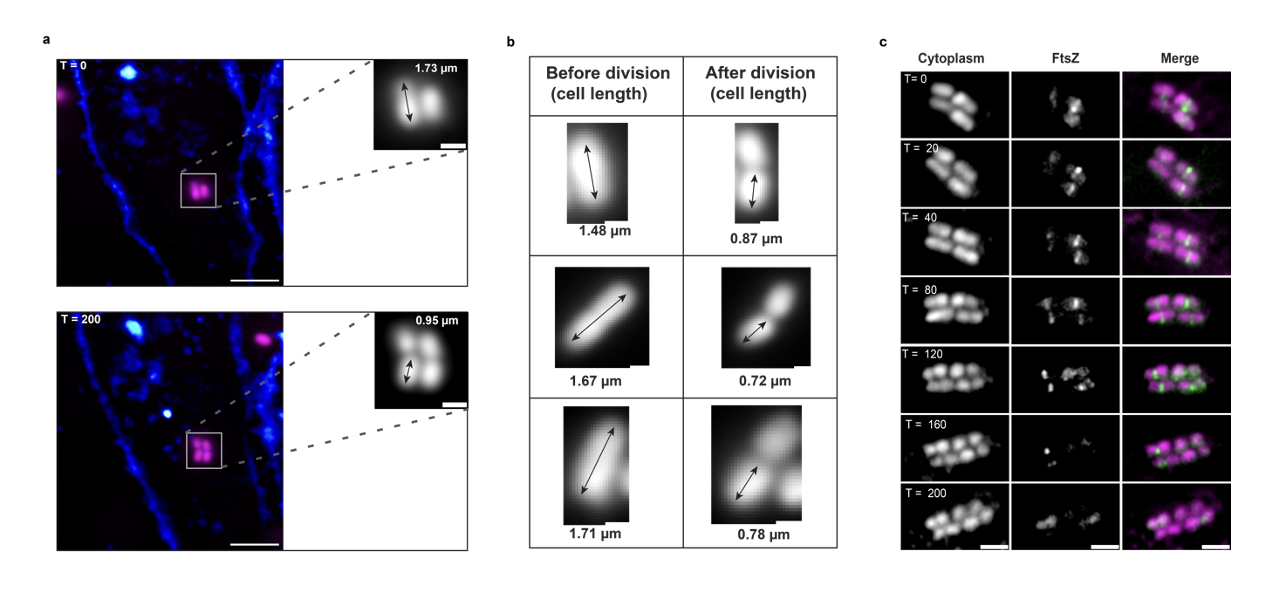

Supplement: S5 Fig — a, Representative images from a time-lapse sequence of PD07i human epithelial bladder cells (membranes in blue) challenged with MS2027 expressing mCherry (pseudo colored magenta) in the cytoplasm; cells before (T = 0) and after division (T = 200). b, Images of intracellular MS2027 cells before and after division across different PD07i human epithelial bladder cells from three separate infections. Example cell lengths of intracellular MS2027 cells before (rods) and after division (coccobacilli) are noted in the table. c, Still images from a time-lapse sequence of intracellular MS2027 cells expressing FtsZ-mCitrine (pseudo coloured green), and mCherry in the cytoplasm. Times (T) are shown in minutes. Scale bars: a = 5 µm (insets 1 µm), b = 0.5 µm and c = 2 µm. (PNG) [file pbio.3003366.s005.png]

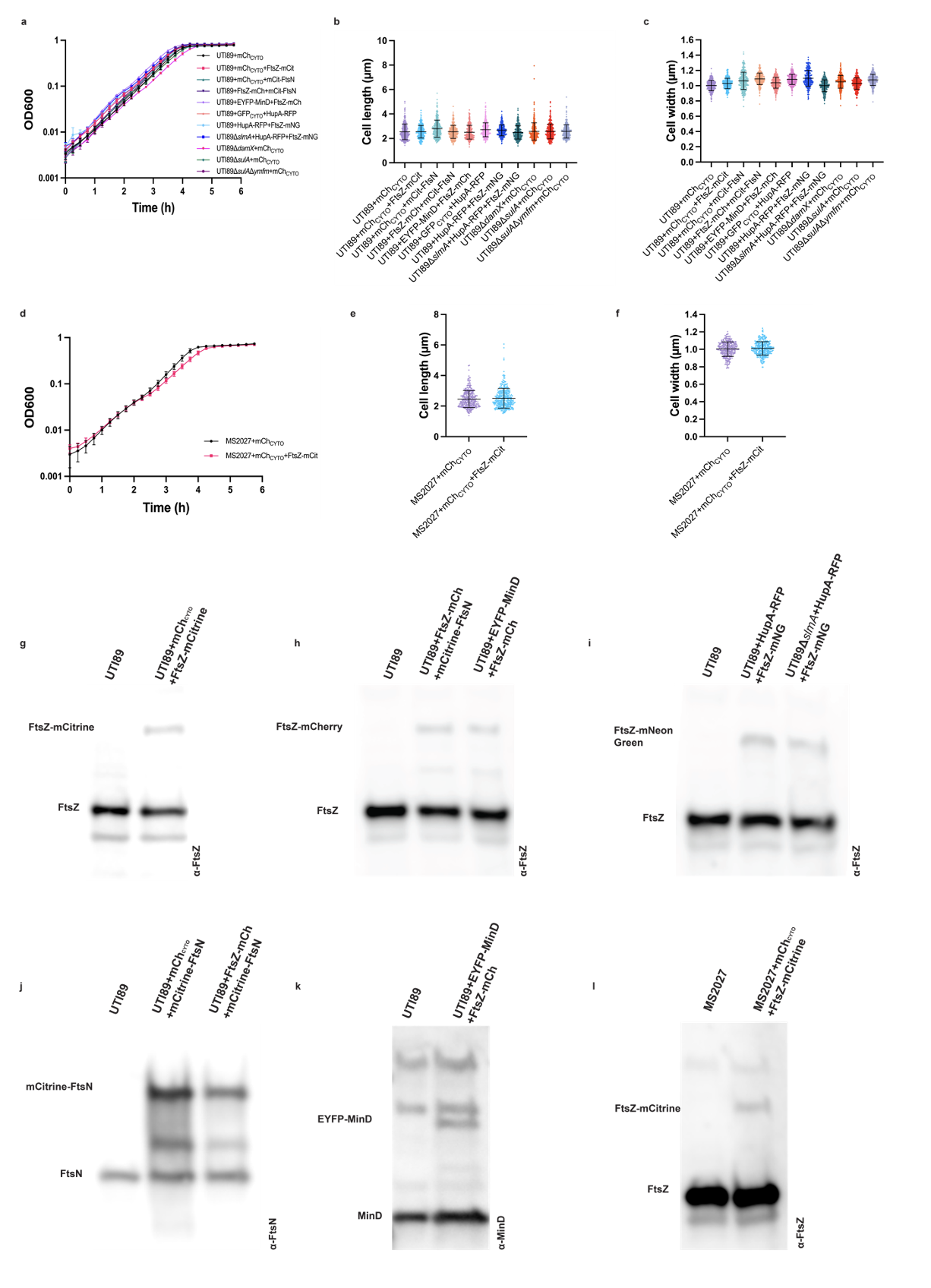

Supplement: S6 Fig — a, Growth curves of UTI89 strains used in the study over a 6h growth period in EpiLife media. b, c, Average cell lengths (b) and widths (c) of UTI89 strains grown in EpiLife media in petri-dishes at 37 °C in the presence of 5% CO2. d, Growth curves of MS2027 strains used in the study over a 6 h period in EpiLife media. e, f, Average cell length (e) and width (f) of MS2027 strains grown in EpiLifemedia in petri-dishes EpiLife media at 37 °C in the presence of 5% CO2. All growth curves include data from 3 biological replicates and ncell = 300 for lengths and widths measurements. Midline represents average, and whiskers indicate S.D. g–I, Quantitative western blots of FtsZ-mCitrine, FtsZ-mCherry, FtsZ-mNeonGreen, mCitrine-FtsN, and EYFP-MinD production levels in UTI89 and MS2027 cells. FtsZ-mCitrine in (g) UTI89, and in (I) MS2027 was produced at 13.5(±5.8) % and 7.2(±3.1) % of the total cellular FtsZ, respectively. (h) FtsZ-mCherry in the UTI89 + mCitrine-FtsN and in UTI89 + EYFP-MinD strains was produced at 16.8(±8.3) % and 18.5(±7.9) % of the total cellular FtsZ respectively. (i) FtsZ-mNeonGreen in the UTI89 + HupA-RFP and in UTI89ΔslmA + HupA-RFP strains was produced to 18(±2.6) % and 17.4(±3.1) % of the total FtsZ. (j) mCitrine-FtsN in UTI89 + mChCYTO and UTI89 + FtsZ-mCherry strains was produced to approximately 142(±14.5) % and 118.7(±1.5) % of the native FtsN production, respectively. Overall, these values for FP-FtsN and FtsZ-FP have previously been shown not interfere with growth and division during vegetative growth in rich media. (k) EYFP-MinD production in the UTI89 + FtsZ-mCherry strain showed that EYFPMinD was produced at approximately 28(±6.4) % of the total cellular MinD. Note some unspecific binding of the anti-MinD antisera. Collectively, these results show that the fluorescent protein fusions were not heavily overexpressed in any of the strains used in this study. All percentages (± S.d.) are based on three independent experiments. mCh, mCherry; mCi [file pbio.3003366.s006.png]

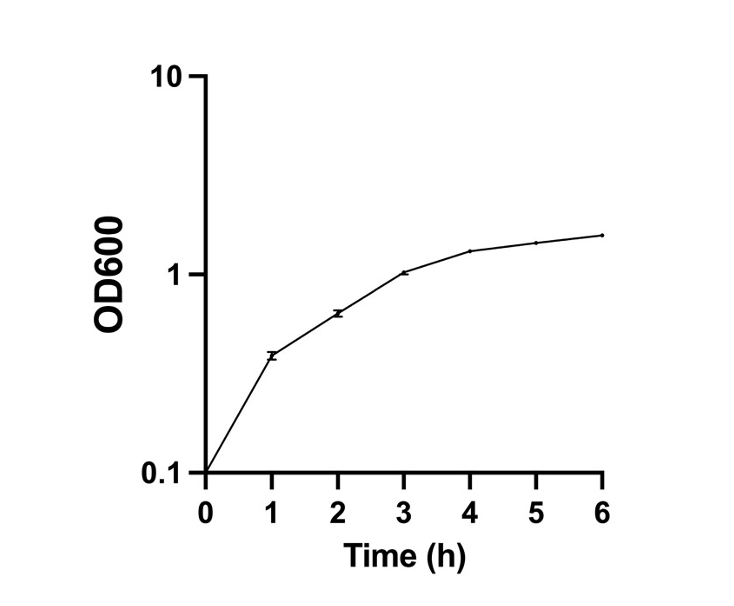

Supplement: S7 Fig — Average doubling time: 31 ± 1 min (n = 3). The data underlying this figure can be found in S1 Data. (PNG) [file pbio.3003366.s007.png]

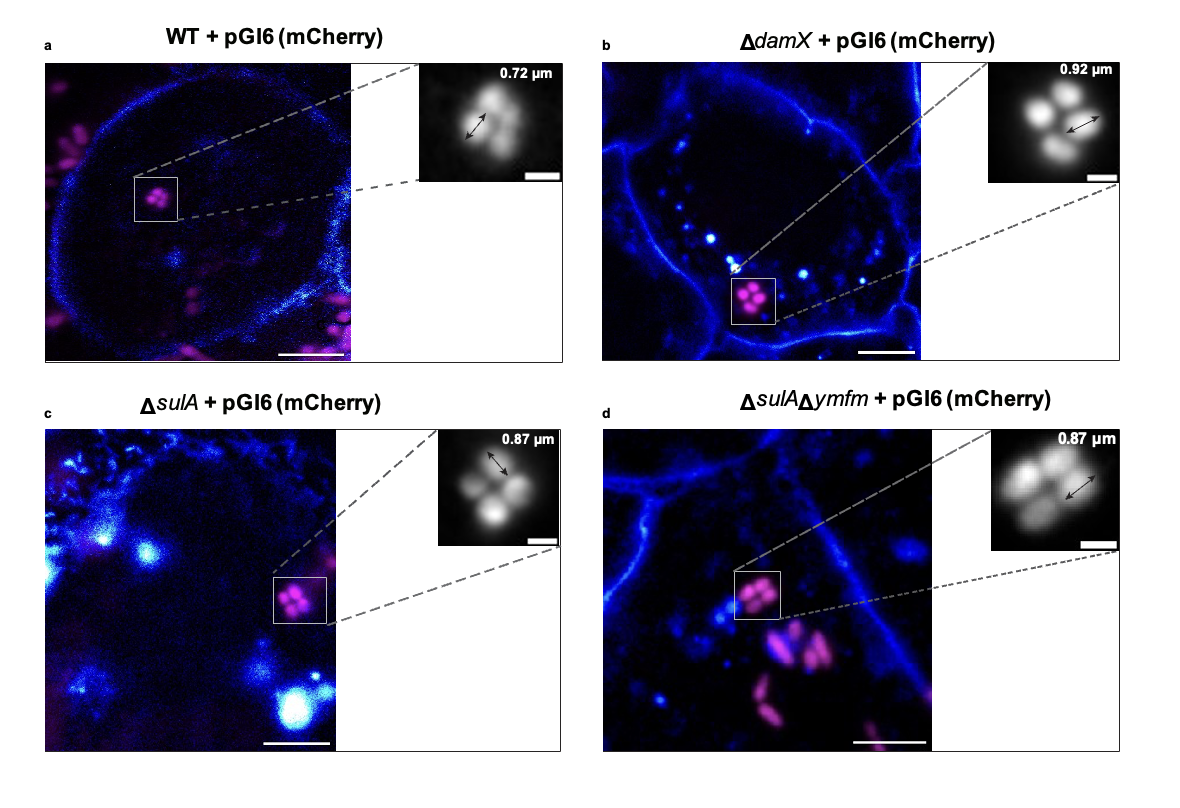

Supplement: S8 Fig — Representative images of PD07i human epithelial bladder cells (membrane in blue) challenged with a, WT UTI89, b, UTI89ΔdamX, c, UTI89ΔsulA, and d, UTI89ΔsulAΔymfM. All strains expressed mCherry from pGI6 in the cytoplasm as a volume marker (pseudo-coloured magenta). Example coccobacilli lengths of parental UTI89 and mutant strains after division are noted in the insets. Scale bars a–d = 5 µm (insets 1 µm). (PNG) [file pbio.3003366.s008.png]

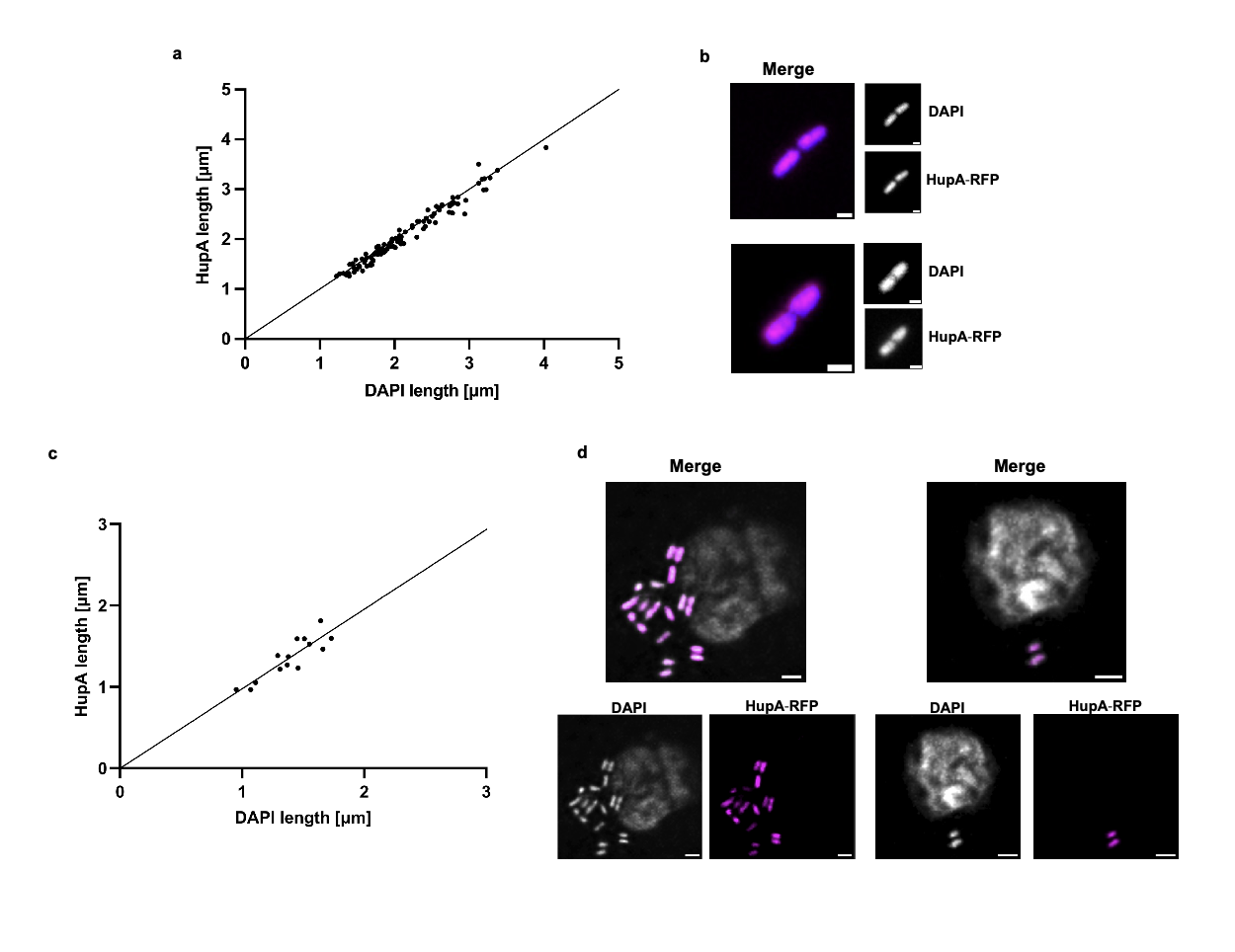

Supplement: S9 Fig — Representative images of nondividing (upper panel) and dividing (lower panel) extracellular UTI89 cells stained with DAPI (blue) and expressing HupA-RFP (pseudo-coloured magenta) (b), and PD07i human epithelial bladder cells challenged with UTI89 expressing HupA-RFP (d). DAPI stained nucleus and intracellular UTI89 cells are shown in gray (pseudo-coloured), and HupA-RFP expression in magenta (pseudo-coloured). n = 103 (a), n = 14 (c). Scale bars (b) = 1 µm, (d) = 2 µm. The data underlying this figure can be found in S1 Data. (PNG) [file pbio.3003366.s009.png]

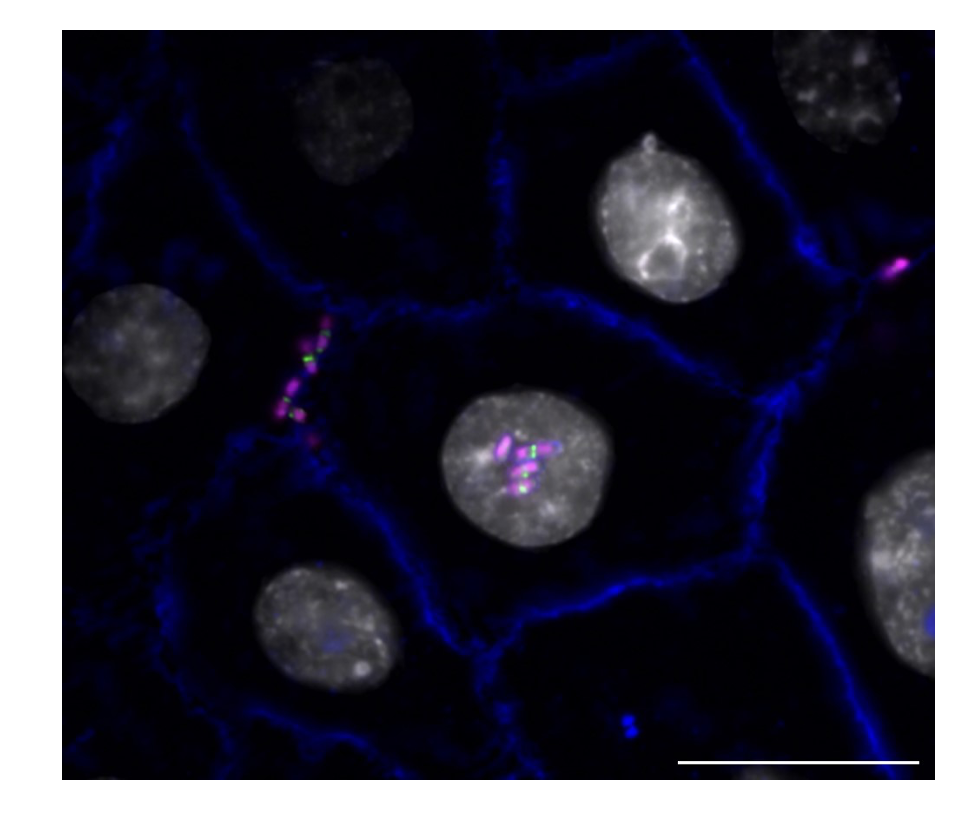

Supplement: S10 Fig — PD07i membranes in blue, nuclei in gray, HupA (pseudo coloured magenta) and FtsZ in green. Scale bar 20 µm. (PNG) [file pbio.3003366.s010.png]

Uncropped western blots:

Blot:

Fig. S6g

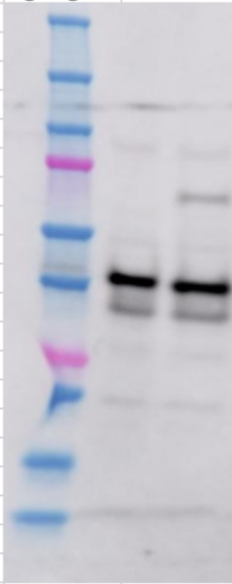

Fig. S6h

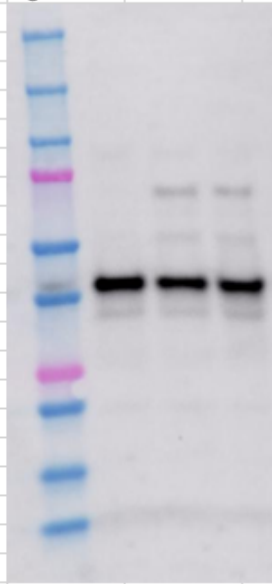

Fig. S6i

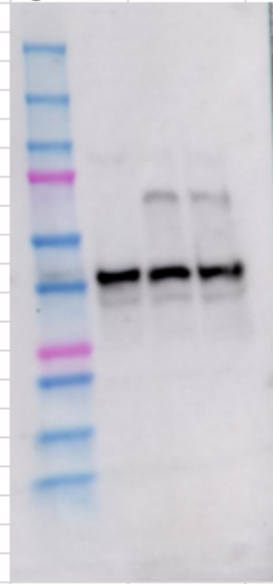

Fig. S6j

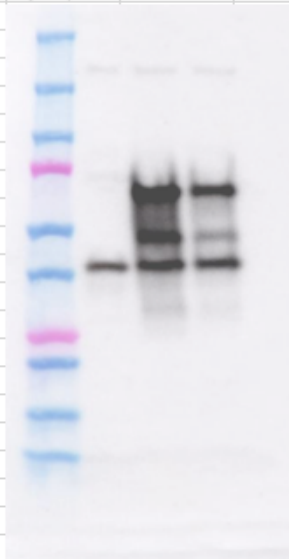

Fig. S6k

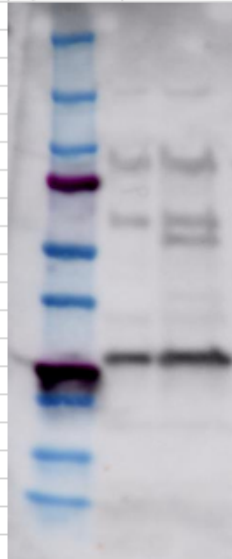

Fig. S6l

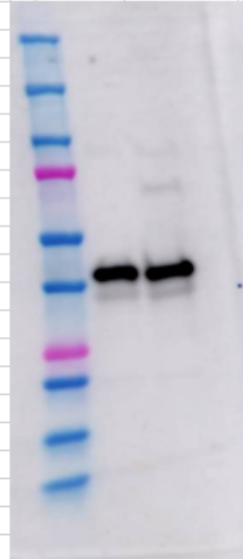

Supplement: S1 Raw Images — (PDF) [file pbio.3003366.s021.pdf]
