# Supplementary material for: Uropathogenic Escherichia coli proliferate as a coccoid morphotype inside human host cells
Source: PLoS Biol. 2025 Sep 3;23(9):e3003366. doi: 10.1371/journal.pbio.3003366 (PMC12407437; doi:10.1371/journal.pbio.3003366)
Supplement: S1 Table — (PDF) [file pbio.3003366.s011.pdf]

***S1 Table**: Bacterial strains and plasmids used in the study.*

Strain	Plasmid(s)	Fluorescent protein(s)	Inducer	Source (ref)	Reference to Figure
UTI89	pGI6	mCherry^{CYTO}	-	96	Fig.1, Supp. Fig 1, Supp. Fig 3, Supp. Fig 6
UTI89	pGI6 / pHC054	mCherry^{CYTO}/ FtsZ-mCitrine	IPTG	96,97	Fig., Supp. Fig 1
UTI89	pGI6 / pHC004	mCherry^{CYTO}/ mCitrine-FtsN	Rhamnose	47,96	Fig.1
UTI89	pMP11 / pHC004	FtsZ-mCherry/ mCitrine-FtsN	Rhamnose	This Study/47	Fig.1
UTI89	pSR-4	EYFP-MinD	IPTG	63	Fig.2
UTI89	pMP11 / pSR-4	FtsZ-mCherry/ EYFP-MinD	IPTG	This Study /63	Fig.2
UTI89	pGI5 / pSTC011	msfGFP^{CYTO}/ HupA-RFP	IPTG	94	Fig.2
UTI89	pMP7 / pSTC011	FtsZmNeonGreen/ HupA-RFP	IPTG	This Study/94	Fig.2, Supp. Fig 7
UTI89Δ*slmA*	pMP7 / pSTC011	FtsZmNeonGreen/ HupA-RFP	IPTG	This Study/94	Fig.2
UTI89Δ*sulA*	pGI6	mCherry^{CYTO}	-	96	Supp. Fig 6
UTI89Δ*sulA* Δ*ymfm*	pGI6	mCherry^{CYTO}	-	96	Supp. Fig 6
UTI89Δ*damx*	pGI6	mCherry^{CYTO}	-	96	Supp. Fig 6
MS2027	pGI6	mCherry^{CYTO}	-	96	Supp. Fig 4
MS2027	pGI6 / pHC054	mCherry^{CYTO}/ FtsZ-mCitrine	-	96,97	Supp. Fig 4
